# Advances in Hepatitis E Virus Biology and Pathogenesis

**DOI:** 10.3390/v13020267

**Published:** 2021-02-09

**Authors:** Shaoli Lin, Yan-Jin Zhang

**Affiliations:** Molecular Virology Laboratory, VA-MD College of Veterinary Medicine and Maryland Pathogen Research Institute, University of Maryland, College Park, MD 20742, USA; lsl1990@umd.edu

**Keywords:** Hepatitis E virus (HEV), virology, life cycle, pathogenesis, virus-cell interactions, Hepatitis E in pregnancy

## Abstract

Hepatitis E virus (HEV) is one of the causative agents for liver inflammation across the world. HEV is a positive-sense single-stranded RNA virus. Human HEV strains mainly belong to four major genotypes in the genus *Orthohepevirus A*, family *Hepeviridae*. Among the four genotypes, genotype 1 and 2 are obligate human pathogens, and genotype 3 and 4 cause zoonotic infections. HEV infection with genotype 1 and 2 mainly presents as acute and self-limiting hepatitis in young adults. However, HEV infection of pregnant women with genotype 1 strains can be exacerbated to fulminant hepatitis, resulting in a high rate of case fatality. As pregnant women maintain the balance of maternal-fetal tolerance and effective immunity against invading pathogens, HEV infection with genotype 1 might dysregulate the balance and cause the adverse outcome. Furthermore, HEV infection with genotype 3 can be chronic in immunocompromised patients, with rapid progression, which has been a challenge since it was reported years ago. The virus has a complex interaction with the host cells in downregulating antiviral factors and recruiting elements to generate a conducive environment of replication. The virus-cell interactions at an early stage might determine the consequence of the infection. In this review, advances in HEV virology, viral life cycle, viral interference with the immune response, and the pathogenesis in pregnant women are discussed, and perspectives on these aspects are presented.

## 1. Introduction

Hepatitis E virus (HEV) is one of the causative agents of viral liver infections. It is estimated that about 20 million HEV infections occur worldwide annually, with 3.3 million symptomatic cases of HEV infection and approximately 44,000 deaths [1]. The virus is usually transmitted via the gastrointestinal route from contaminated water or food of animal origin. Currently, at least four genotypes of HEV are known to infect humans [2]. Genotypes 1 and 2 viruses cause acute hepatitis in the general population, with a case fatality rate of 0.5%–3%. Infection of pregnant women with genotype 1 HEV may cause acute liver failure, leading to a case fatality rate of up to 30% (reviewed in [3]). Genotype 3 strains are the main causes of chronic HEV infection in elderly or immunocompromised patients, with a high risk of progressing to liver cirrhosis [3]. Sporadic cases of chronic infection by genotype 4 are also reported. The exact mechanisms for the different disease outcomes by genotypes are still unknown; however, recent research has provided some clues. The virus-cell interactions are complex and may determine the outcome of infection. A section below is dedicated to trying to shed light on this front, although there are many unknowns.

HEV is a positive-sense, single-stranded RNA virus with an icosahedral capsid. Two forms of HEV virions are known: quasi-enveloped and non-enveloped. The peak density of the naked, non-enveloped particles is 1.27 g/mL, while the quasi-enveloped particles have a density of 1.15 g/mL [4,5]. The HEV genome is about 7.2 kb and encodes three open reading frames (ORFs). An additional ORF, ORF4, is found only in genotype 1 strains [6]. ORF1 encodes the non-structural proteins for genome replication, ORF2 encodes the capsid protein, and ORF3 encodes a small multifunctional protein.

Contemporary studies have provided informative insights into the HEV life cycle. HEV was once considered as a non-enveloped virus, but quasi-enveloped virions are found in the blood circulation and cultured cells [7]. The lipid membrane shields the virions from neutralizing antibodies. There are multiple forms of ORF2 product in patient serum and cultured cells: the capsid protein associated with virions and the soluble protein glycosylated and secreted. These results reveal an interesting feature of the capsid protein biogenesis.

HEV infection interferes with cell signaling and evades the antiviral responses of innate immunity. HEV inhibits the induction of type I interferons [8] and can persist in the presence of type III interferons [9]. The viral proteins encoded by ORF1, 2, and 3 interact with cellular partners to downregulate antiviral factors and recruit elements to generate a conducive environment for HEV replication.

## 2. HEV Taxonomy and Distribution

HEV strains are classified into two genera: *Orthohepevirus* and *Piscihepevirus*, in the family *Hepeviridae* [10]. The genus *Orthohepevirus* contains four species, namely A, B, C, and D, which infect humans and animals. *Piscihepevirus* is isolated only from salmonid fish in North America so far. *Orthohepevirus A* consists of the previously known genotype 1–4 and the newly recognized genotype 5–8. Genotype 1 and 2 are restricted to humans [11]; genotype 3 and 4 are zoonotic and have been detected in a wide spectrum of hosts, including monkey, pig, sheep, cow, wild boar, deer, rabbit, and mongoose [12,13,14,15,16,17,18,19,20,21,22]; genotype 5 and 6 are reported to infect only wild boars [2], with genotype 5 having the potential for zoonotic infection [23]; genotype 7 and 8 are isolated from camels, with a sole case report of human infection from genotype 7 [23,24]. *Orthohepevirus B* consists of avian HEV [2]. *Orthohepevirus C* consists of rat and ferret HEV, while *Orthohepevirus D* contains only bat HEV [2]. A rat HEV strain of *Orthohepevirus C* was found in a patient with persistent hepatitis after liver transplantation, suggesting the zoonotic potential of this species [25]. The genomic variability and potential risk of cross-species infection of *Orthohepevirus C* strains have been reviewed elsewhere [26,27]. Along with the isolation of HEV from more wild and domestic animals, an evolution of the virus taxonomy is expected in the future.

The regional prevalence of HEV genotypes is varied. Among the four major genotypes that infect humans, genotype 1 is mainly distributed in South Asia and sub-Saharan Africa [28,29], where the infection is predominantly due to poor sanitation and contaminated drinking water. Genotype 2 was initially discovered in Mexico and later found in Africa [30,31,32,33]. Genotype 3 contributes to pockets of hepatitis E cases in industrialized countries [34,35,36] and also widely spreads in some developing countries in Latin America [37,38] and China [39]. The most frequent route of transmission of genotype 3 HEV in humans is the consumption of HEV-contaminated uncooked/undercooked pork or sausage [40,41]. Genotype 4 is most prevalent in China but also detected in other countries, such as South Korea, Japan, and France [42,43,44]. Like genotype 3, genotype 4 HEV can be transmitted via contaminated food. The co-infection of genotypes 3 and 4 in patients with acute hepatitis was reported in Japan [45].

In addition to the gastrointestinal route, HEV has been demonstrated to be transmitted via blood transfusion in some countries [46,47]. Genotype 1 HEV has also been detected in blood donors in India [48]. 

Despite the genetic difference, the four genotypes belong to a single serotype. The antibody against the neutralization epitopes of the capsid protein of HEV genotype 3 can neutralize the other different geographic HEV strains in genotype 1 and 2 [49]. Commercial enzyme immunoassays and rapid immunochromatographic kits based on genotype 1 HEV ORF2/ORF3 antigens can detect the presence of IgM or IgG antibodies induced by the four major genotypes of HEV [50,51].

## 3. HEV Genome and its Encoded Proteins

**Figure 1:**
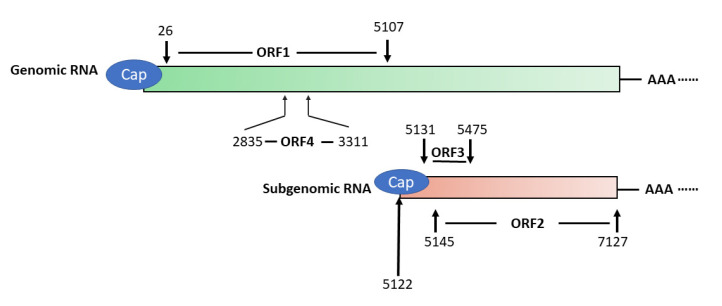
Schematic illustration of Hepatitis E virus (HEV) genomic and sub-genomic RNA. The genome organization of genotype 1 HEV strain Sar55 (GenBank accession number: AF444002) is shown. The numbers above and below the boxes denote the nucleotide position in the genome. ORF1 spans nucleotide (nt) 26–5107 and encodes a polyprotein of 1694 amino acids (aa) in length, which contains several putative domains. ORF2 (nt5145–7127) and ORF3 (nt5131–5475) are translated from the sub-genomic RNA, and ORF4 is only produced under endoplasmic reticulum (ER) stress and solely in genotype 1 HEV.

HEV has a 7.2 kb positive-sense, single-stranded RNA genome that has a 5′ cap and 3′ poly(A) tail [52] (Figure 1). The genome encodes three partially overlapping ORFs, namely ORF1, ORF2, and ORF3. A newly-identified ORF4 that is embedded in ORF1 is found in the genomes of only genotype 1 strains [6]. Also, the virus produces a 2.2 kb sub-genomic RNA in infected cells [53]. Transcription of the sub-genomic RNA in genotype 1, 3, and 4 HEV starts with the common starting sequence of 5′GC [54]. Both ORF2 and ORF3 are translated from the sub-genomic RNA [53]. The abundance of sub-genomic RNA is much higher than the genomic RNA in HEV-infected cells, indicating that the expression of ORF2 and ORF3 products must be higher [9].

### 3.1. ORF1

**Figure 2:**
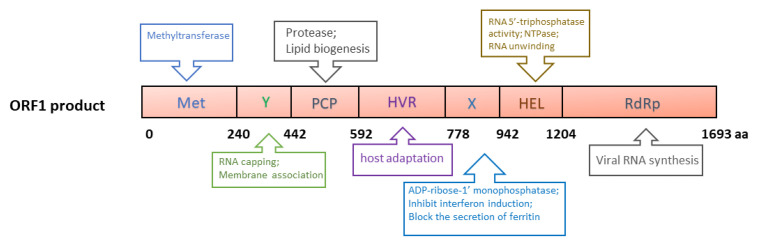
Schematic illustrations of the putative domains in the ORF1-derived polyprotein. Met: Methyltransferase domain; Y: Y domain; PCP: papain-like cysteine protease; HVR: hypervariable region; X: X domain; Hel: helicase; RdRp: RNA-dependent RNA polymerase. The numbers below the box indicate the amino acid residues. The predicted or known functions of the domains are indicated in upper and lower boxes.

ORF1 of genotype 1 HEV is 5082 nucleotides (nt) long and encodes a 190 kDa polyprotein, which contains the following putative domains: methyltransferase domain (Met), Y domain (Y), papain-like cysteine protease (PCP), hypervariable region (HVR), X (macro) domain, helicase domain (Hel) and RNA-dependent RNA polymerase domain (RdRp) (Figure 2) [55]. The polyprotein is putatively processed into several non-structural proteins (NSPs), but this is still debated due to the lack of solid data [56,57]. A plasmid containing ORF1 produces a protein of ~191 kDa in cell-free translation and cleaved products of 78 and 35 kDa in cultured cells [58]. A recent report describes the generation of an HA-tagged full-length HEV replicon with transposon-based technology [59]. The infectious virus was recovered from the replicon with an HA tag within the ORF1 HVR. In the replicon system, only an uncleaved ~190 kDa ORF1 product was detected. Thus, protease processing the ORF1 product remains inconclusive.

The major functions of ORF1 products are predicted to directly facilitate viral RNA synthesis, involving RNA capping [60,61], RNA unwinding [62], tRNA metabolism [63], orienting the viral RNA to replication factories, transcription, and replication [64] (Figure 2). Currently, the mechanism of the involvement of the ORF1 products in viral genome replication remains unclear. However, due to the lack of efficient reagents, such as monoclonal antibodies against the individual domains, and a highly permissive cell culture system, some structural predictions have not been verified experimentally. Details of the ORF1 domains have been reviewed elsewhere [65].

### 3.2. ORF2

**Figure 3:**
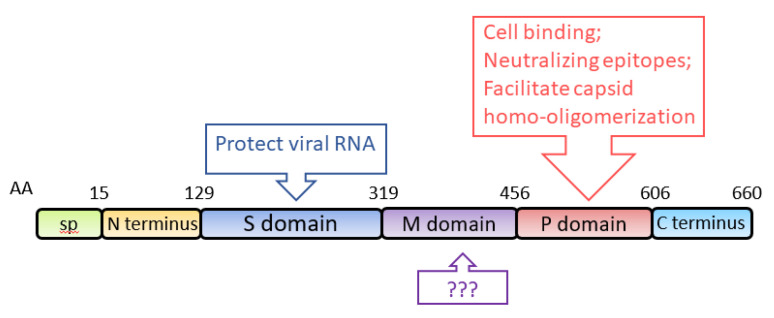
Schematic illustration of the domains of the HEV capsid protein. The numbers above the box indicate amino acid residues of the capsid protein, and the domains are indicated. The known functions of the domains are indicated in upper and lower boxes. SP: signal peptide; S domain: shell domain; M domain: middle domain; P domain: protruding domain.

ORF2 encodes the capsid protein of HEV, with an estimated molecular weight of 72 kDa. When expressed in two insect cell lines (SF9 and Tn5), aa 112-608 self-assemble into virus-like particles (VLPs) [66,67]. Structural analysis shows that the capsid protein of HEV genotype 3 contains an N-terminal domain (aa 1–111), a VLP (aa 112–608), and a C-terminal domain (aa 609–660) [68]. Analysis of the VLP crystal structure at a 3.5-Å resolution shows that it consists of three definite domains, named S (shell), M (middle) and P (protruding), spanning aa 129–319, 320–455, and 456–606, respectively [69] (Figure 3). S domain is the building block of the capsid, exhibiting a “jelly roll” β-barrel fold that forms a tightly closed shell protecting the viral RNA [70]. P domain is responsible for the binding of virions to susceptible cells and contains virus-neutralizing epitopes [69]. Epitopes of several neutralizing monoclonal-antibodies (mAb) against the capsid protein are mapped to the P domain [71,72,73]. Some of the mAb were demonstrated to block HEV infection in rhesus monkeys [71]. Neutralizing epitopes are also found in the M domain and the C-terminal domain [72,74]. P domain also contains the motif for homo-oligomerization of the capsid protein [75]. Deletion of aa 585–610 results in the loss of the oligomerization. Several residues (Y557, T564, V598, A599, L601) within the P domain are crucial for the dimerization of the domain [76]. Because VLPs are an efficient antigen for the detection of HEV-specific immunoglobulins (IgG and IgM) and the predominant carrier of T- and B-cell epitopes [66,77], the VLPs are frequently used for diagnostic ELISA of clinical samples [78,79,80,81].

In cultured cells and patient samples, three forms of the ORF2 product of genotype 3 HEV have been identified: ORF2i (infectious form associated with virions), ORF2g (glycosylated and secreted), and ORF2c (cleaved and secreted) [82]. The first residues of ORF2i, ORF2g, and ORF2c correspond to Leu14, Ser34, and Ser102, respectively [82,83]. ORF2c is likely a cleavage product of ORF2g protein and not a directly translated peptide [83]. Among the three potential glycosylation sites, N1 (137NLS), N2 (310NLT), and N3 (562NTT), of the ORF2 product, only the N1 and N3 sites in the ORF2g/c proteins are glycosylated, whereas the ORF2i is not glycosylated [83]. Further mutation experiments indicated that the N-glycosylation of the ORF2 product does not play any role in the assembly and infectivity of HEV particles. Among the three forms, only ORF2i is packaged into infectious particles. However, all of the three forms can be recognized by HEV antibodies [83].

Another study determined the initiation codons for the secreted form of ORF2 product and the actual capsid protein (Table 1) [84]. The secreted form of ORF2 product (ORF2s) is initiated from the previously presumed start codon and has no association with the viral genome. The actual capsid protein (ORF2c) is initiated from an internal AUG located 15 codons downstream of the first AUG [84]. ORF2s is glycosylated and lacks the binding site of the cellular receptor, but the protein inhibits antibody-mediated neutralization of HEV [84]. In terms of the functional similarity between these two studies, the ORF2s and ORF2c correspond to the ORF2g and ORF2i, respectively, despite the different initiation sites identified. However, this study [84] did not find the cleaved form ORF2c, presumably due to its lower level [83]. These data reveal the complexity of ORF2 genesis and its multifunctional roles in the HEV life cycle.

Intriguingly, the secreted forms of ORF2 product are not involved in the virion packaging. The exact function of the secreted forms is not understood yet. These findings raise a series of questions: how long is the half-life of the secreted protein in the blood circulation? What is the ability of the secreted protein to induce neutralizing antibodies? Whether do the different forms of the ORF2 product contribute to the viral infectivity during HEV infection?

### 3.3. ORF3

ORF3 is a small multifunctional 13 kDa protein (hereinafter called vp13). Its translation starts upstream of ORF2, overlapping with ORF2 by 331 nt in a different frame. The small regulatory viral protein contains a few B-cell epitopes [85,86], but the lymphoproliferative response to the vp13 peptide pool is poor [77].

**Figure 4:**
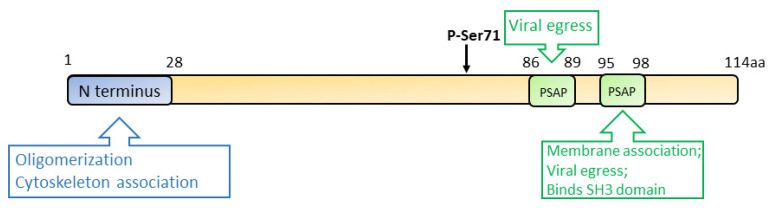
Schematic illustration of the domains of the HEV ORF3 product. The numbers above the box indicate the amino acid residues. The N terminus, phospho-serine site, and PXXP motifs are indicated. Known functions of the motifs are indicated in the upper and lower boxes.

Vp13 is associated with the quasi-enveloped virions, but it dissociates from the naked virus particles after the removal of the envelope [87,88]. Ser71 residue of vp13 is a phosphorylation site that is indispensable for the interaction with the capsid protein [89,90] (Figure 4). However, a later study demonstrated that a mutant HEV lacking the phosphorylation in vp13 replicates in cells and induces viremia in the rhesus monkey, suggesting that vp13 phosphorylation is dispensable for virus replication and infection [91]. While vp13 is not required for virus replication or infection in cultured cells [53], it is needed for infectivity in vivo [91,92]. During HEV replication, vp13 oligomerizes and associates with the membranes through its N-terminal 28 residues [93]. Further study revealed that vp13 is palmitoylated through modification of the N-terminal cysteine residues, assisting its association with membranes and secretion of infectious virions [93]. The N terminal hydrophobic domains of vp13 also mediate its association with microtubules, possibly facilitating the viral egress [94].

During HEV egress, the tumor susceptibility gene 101 (TSG101/ESCRT-I) and the enzymatic activities of Vps4A and Vps4B are required, suggesting the involvement of the multivesicular body (MVB) pathway in the virion release. Two PXXP motifs (aa 86–89 and 95–98) of vp13 are necessary for HEV release [95,96]. Vp13 interacts with TSG101 via one of its PSAP motifs (aa 95–98) to facilitate the virus release [97]. Aside from interacting with the MVB pathway, vp13 is also demonstrated to be a class I viroporin, forming an ion channel for the viral release [98]. Mutations of the two PXXP motifs of vp13 abrogate the virus release but do not affect the ion channel activity. Notably, the mutations of vp13 (CCC11-13AAA and IFI59-61AAA) not only abolish the viral release but alter the protein subcellular location, implying the involvement of the two motifs in the ion channel activity and viral release. The data suggest another mechanism of viral egress other than the MVB pathway [98].

Vp13 also plays a role in disturbing cellular signaling. Vp13 of genotype 1 and 3 HEV can elevate retinoic acid inducible gene 1 (RIG-I) signaling by enhancing its stability, while the vp13 from genotype 2 and 4 reduce the signaling [99]. Another study also shows the vp13 of genotype 4 downregulates the induction of type I interferons (IFNs) via degrading IRF7 [100]. The variation between vp13 of the different genotypes is intriguing and needs further investigation.

### 3.4. ORF4

ORF4 is derived from ORF1 and located in a +1 frame of genotype 1 strains only, spanning nt 2835–3311. ORF4 is produced only under endoplasmic reticulum (ER) stress [6]. The expression of the ORF4 protein is cap-independent and internal initiation-mediated. This protein functions to stimulate viral polymerase activity [6].

For further details of all the viral proteins of HEV, one can refer to this review [56,101]. Although the structures and characteristics of some viral proteins have been defined, their exact functions in HEV pathogenesis largely remain unclear. To better understand the functions of the viral proteins, highly specific monoclonal antibodies are needed.

## 4. HEV Life Cycle

**Figure 5:**
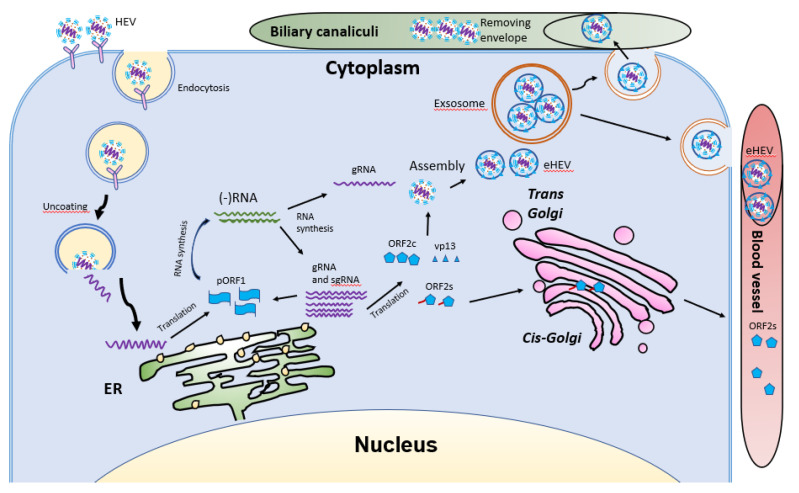
Schematic illustrations of the HEV life cycle. HEV particles bind the cellular receptor and enter cells via receptor-mediated endocytosis. After uncoating, the viral genomic RNA is released and serves as mRNA for ORF1 translation. RdRp synthesizes negative-sense intermediate RNA, followed by synthesis of genomic RNA (gRNA) and sub-genomic RNA (sgRNA). Structural proteins are translated, followed by assembly and egress. The eHEV is released into biliary canaliculi, where the envelope is removed, and naked virions are released into intestines and excreted in feces. The eHEV is also released into the blood vessels. The ORF2s is glycosylated and secreted, followed by circulation in the bloodstream.

HEV virions are conventionally viewed as non-enveloped particles, ranging from 20–40 nm in diameter. However, HEV particles from cultured cells and serum samples are found to be quasi-enveloped. HEV virions from cultured cells and monkey feces have different densities and sedimentation coefficients. The virions from cell culture supernatant possess lipid and vp13, while those from feces do not [88]. The non-enveloped virions are enterically transmitted and possibly enter the bloodstream after the first round of replication in an unknown cell type in the gut. The virions then reach hepatocytes from the bloodstream. The enveloped virions in the uncooked/undercooked meat may also be enterically transmitted and the envelop is presumably removed during passage in the gastrointestinal tract. The enveloped virions that are transmitted via blood reach hepatocytes and extra-hepatic target cells from the bloodstream. The HEV cell entry has been reviewed elsewhere [102]. Non-enveloped virions require heparan sulfate proteoglycan (HSPG) for attachment to target cells, but the enveloped virions (eHEV) attach to the cells independent of HSPG [4]. Both types of virions enter the cells through clathrin-mediated and dynamin-2-dependent endocytosis (Figure 5). During the endocytosis of HEV, low pH is required but not enough for the uncoating of eHEV [4]. However, the mechanism of uncoating is not well understood.

After uncoating, the positive-sense RNA genome is released into the cytosol and serves as the template for the translation of ORF1. The 7-methylguanosine cap structure at the 5′UTR of the HEV genome recruits the 40S ribosomal subunit to initiate cap-dependent translation. Once produced, RdRp will initiate transcription of the viral genomic RNA by binding to its 3′UTR to produce the negative-sense intermediate RNA [64]. This intermediate RNA serves as the template for the synthesis of progeny positive-sense viral genomes. HEV replication requires Golgi-specific brefeldin A-resistant guanine nucleotide exchange factor 1 (GBF1) [101,103]. The ubiquitin-proteasome system also contributes to HEV replication as inhibition of the system abolishes the viral replication [103,104]. For viral encapsidation and assembly, the capsid protein interacts with a 76nt region specifically in the 5′ end of the HEV genome [104]. The N-terminal 111 amino acid residues of the capsid protein appear to not be involved in the interaction.

Following the assembly, the viral progeny are transported by multivesicular bodies and released by the cellular exosomal pathway [105]. The HEV assembly and release are reviewed elsewhere [106]. Most infectious HEV particles in the form of eHEV are released from the apical side of the hepatocytes into the biliary canaliculi, where the eHEV are converted to non-enveloped particles by the detergent in the bile (Figure 5). A small portion of eHEV particles is released into the blood via the basolateral side of the hepatocytes. After the virion release, only non-enveloped HEV can be detected in bile and feces, but in blood and urine virions are likely to be enveloped [107,108]. The eHEV envelope possesses the trans-Golgi network protein 2 (TGOLN2), one of the markers of the trans-Golgi network, suggesting that the membrane-associated HEV particles are derived from the intracellular membrane but not the cell surface [7]. The eHEV particles are shown to contain the capsid and vp13 proteins, while the non-enveloped particles only contain the capsid protein. It is important to note that, due to the surrounding of an envelope, the eHEV cannot be neutralized by capsid-specific monoclonal antibodies. The absence of vp13 in the naked virions suggests that this protein is only required for the release but not the entry of the virus. In addition to the reduced recognition by antibodies, the attachment efficiency of the eHEV virions to hepatocytes is one-tenth that of the naked virions [4]. Conversely, the infectivity of the eHEV particles is shown to increase after the removal of the lipid layer. Considering the gastrointestinal transmission route for HEV, the eHEV is the source of naked virions and may contribute to dissemination.

During the formation of eHEV in the cells, the virus acquires the envelope mainly from the intracellular membrane. During this process, how the virus alters the process of the host fatty acid is still unknown. The presence of the lipid envelope seems to assist the virus in evading neutralizing antibodies. However, the shorter duration of viremia than the presence of non-enveloped virions in feces suggests that the eHEV might have a minor role in HEV transmission. The HEV life cycle has been reviewed elsewhere [109].

HEV infection can evoke a series of physiological and immunological alterations in the host. Viremia in acute hepatitis E patients normally lasts for one month, during which the anti-HEV IgM is the major antibody produced [110,111]. Anti-HEV IgM can still be detected in about 40% of patients until 12 months. Viral RNA can become undetectable when the patients come to clinical attention (reviewed in [110]). However, with the newly developed pan-genotypic PCR-based assay that is more sensitive than the commercial kits and detects the entire spectrum of the genotypes in *Orthohepevirus A* [112], detection and quantification of HEV in clinical samples could be significantly improved. Thus, HEV infection might be less underestimated, and viral RNA can be detectable in clinical settings with a better possibility. IgG production usually peaks at 20 weeks post-infection and can be detected after two years, in around 37% of cases. Immunohistochemical analysis of liver biopsies shows the infiltration of activated CD8+ T cells, which may lead to liver damage during acute liver failure [113,114].

Both innate and adaptive immune responses are needed to clear the virus. HEV has evolved a series of strategies to evade immune responses. HEV-specific T-cell responses in patients with chronic hepatitis E are absent but detectable after viral clearance, suggesting an association with impaired T-cell immune response [115,116]. Robust HEV-specific T cell responses predominantly targeting the capsid protein are present during acute infection, while low-level response is seen in immunosuppressed patients [117]. A recent study defines the T cell receptors that target HEV-specific CD8+ T cell epitopes in HEV helicase and RdRp, which are explored for immunotherapy of chronic hepatitis E [118].

## 5. Manipulation of Host Factors by HEV for Its Replication

### 5.1. Interference with Innate Immune Response

Innate immunity includes immune cells in the first line of defense, such as macrophages and dendritic cells, and soluble factors, such as interferons (IFNs) [116,119]. IFNs play vital roles in early antiviral defense. For efficient replication, HEV needs to antagonize IFN induction and downstream signaling. Strains of different virulence may have a variable antagonizing effect on IFNs and the effect might contribute to the different disease manifestations. There are three types of IFNs: type I, type II, and type III. Although all of the three types of IFN exert inhibition of HEV replication, the effect is less potent than for hepatitis C virus (HCV) restriction [119]. To survive the innate immune defense, HEV also significantly attenuates the production of downstream antiviral IFN-stimulated genes (ISGs) [119]. The HEV interference with the IFN signaling reported by previous studies is described below.

#### 5.1.1. Interplay with Type I IFNs

Type I IFNs include IFN-α, IFN-β, IFN-κ, IFN-ω, and IFN-ν [120,121]. IFN-α and IFN-β play antiviral roles by inducing the production of many IFN-stimulated genes (ISGs) [122], which arm cells against virus infection and activate the immune cells, such as macrophages and dendritic cells [123]. Induction of IFN production in the cells is mainly through pattern recognition receptors (PRRs), such as retinoic-acid-inducible gene I (RIG-I)-like receptors (RLR) and Toll-like receptors (TLRs) [124]. Activation of RIG-I leads to the conversion of mitochondrial antiviral signaling protein (MAVS) into prion-like polymers [124,125]. The polymerized MAVS then bind several E3 ligases, such as TNF receptor-associated factors 2, 3, and 6 (TRAF2, 3, and 6) [126], followed by recruitment and activation of the serine/threonine-protein kinase TANK-binding kinase 1 (TBK1). Activated TBK1 then phosphorylates MAVS, leading to the recruitment of IFN regulatory factor 3 (IRF3) to MAVS for phosphorylation. Upon phosphorylation, IRF3 is dimerized, dissociated from MAVS, and translocated into the nucleus to activate the expression of IFNs [127]. RNA helicase DDX3 couples with MAVS to promote the transcription of IFNs. Knockdown of DDX3 reduces IFN production [128,129].

IFNs exert their functions by binding to their receptors on target cells and activating the Janus kinases (JAK)-signal transducer and activator of transcription (STAT) pathway, leading to transcription of a myriad of ISGs [128]. Many ISGs function as restriction factors of virus replication at various steps. In vitro experiments show that type I IFNs present the strongest inhibition of HEV replication [119]. However, immunohistochemistry analysis of the expression of IFN-α and IFN-inducible GTP-binding protein Mx in livers of pigs experimentally infected with swine HEV discovered that the expression of IFN-α and Mx in Kupffer cells, lymphocytes, and hepatocytes was inversely correlated with the number of HEV-infected cells [130], suggesting the virus must have certain strategies to dampen the IFN response. In Huh7.5 cells that have a defective RIG-I, HEV replication efficiency is higher than in HepG2/C3A cells that have intact PRRs for IFN induction. Meanwhile, the reconstitution of RIG-I in Huh7.5 cells significantly restricts the replication of HEV [131], indicating an essential role of type I IFNs in the inhibition of HEV replication.

HEV antagonizes the production of both type I and III IFNs [8,132]. The ORF1-derived X domain and PCP mediate blocking of the phosphorylation of IRF3 and the de-ubiquitination of RIG-I and TBK-1, respectively [8]. However, HEV does not cleave MAVS to inhibit RIG-I signaling [9]. The capsid proteins of both genotypes 1 and 3 impair the production of type I IFNs [132]. Mechanistically, the capsid protein blocks the phosphorylation of IRF3 via interaction with the multiprotein complex consisting of MAVS, TBK1, and IRF3. The N-terminal domain of the capsid protein appears to be responsible for the inhibition of IRF3 activation. Further study shows that the arginine-rich-motif in the N-terminal domain is essential for inhibition as mutations of the arginine residues abolished the blockage. As the capsid protein is produced at a much higher level than the ORF1 products and vp13 in infected cells, the capsid protein is expected to play a major role in antagonizing interferon production. The capsid protein interacts with RNA helicase DDX3 [133]. The DDX3 C-terminal domain is found to interact with the capsid protein. Knockdown of DDX3 compromises the capsid protein-mediated blockage of interferon induction. These results provide further insight into HEV interference with innate immunity.

#### 5.1.2. Induction of Type II IFN

The type II IFN has only one subtype, IFN-γ. IFN-γ is produced by NK cells, NKT cells, CD4+ T, and CD8+ T cells [134]. IFN-γ stimulates undifferentiated CD4+ T cells to differentiate into Th1 cells and helps to activate NK or NKT cells. In peripheral blood mononuclear cells (PBMC) from patients with acute HEV infection, stimulation with HEV capsid protein induces the elevation of IFN-γ level but does not change the proportion of CD4+ T and CD8+ T cells, which indicates that HEV infection sensitizes NKT cells to produce IFN-γ [135]. Meanwhile, in HEV-infected pregnant women, IFN-γ, TNF-α, and IL-6 are elevated compared to non-pregnant women [136]. Compared to type I and III IFNs, IFN-γ only exerts moderate inhibition of HEV replication, according to an in vitro experiment [119].

#### 5.1.3. Interplay with Type III IFNs

The type III IFNs are composed of IFN-λ1, -λ2, -λ3 and -λ4 [137,138]. HEV can survive in the presence of type III IFNs [9,139]. Different from type I IFNs, whose receptor is distributed on all nucleated cells, the receptor of IFN-λ is constricted to epithelial and immune cells, such as neutrophils and NK cells [140,141]. Production of type III IFNs is similar to, but has a minor difference from, type I IFNs. Type I IFNs are induced via the mitochondrial-associated MAVS, while type III IFNs are produced via activation of the peroxisome-associated MAVS [142,143]. IRF1, IRF3, and NF-κB mediate the signaling leading to IFN-λ production from the peroxisome-bound MAVS [142,143]. The signaling cascades downstream of the IFN-λ receptor complex are very similar to those of type I IFNs and are transmitted by the JAK-STAT pathway [144]. IFN-λ protects epithelial layers and mucosal barriers from the invasion of pathogens [145] and regulates adaptive immunity through activating dendritic cells [146].

HEV induces type III IFNs via RIG-I and MDA5 and elevation of the two PRRs [9]. However, elevated RIG-I or MDA5 do not enhance type I IFN production, possibly due to the inhibitory roles of HEV viral proteins [8,132]. Although type III IFNs can suppress the replication of HEV to a certain extent, the virus can still survive and persist in the presence of low-level type III IFNs. The continuous activation of IFN signaling renders the infected cells resistant to extra exogenous IFN treatment, probably due to the consistently activated JAK/STAT pathway [9]. HEV inhibits poly(I:C)-induced type III IFNs, albeit weaker than the inhibition of type I IFNs [132].

### 5.2. Manipulation of Cellular Factors for a Conducive Environment for Replication

HEV can manipulate cellular proteins to create a conducive environment for replication and to reduce immune response. Liver samples from swine infected with genotype 3 HEV revealed the upregulation of apolipoprotein E [147]. In patients infected with HEV, the triglycerides (TG) and low-density lipoprotein cholesterol (LDL-C) are elevated in the serum, suggesting an imbalance of lipid homeostasis caused by HEV [148]. Golgi brefeldin A resistance factor 1 (GBF1) is a lipid regulator that participates in vesicle transport, Golgi morphogenesis, and lipid droplet metabolism. The depletion of GBF1 impairs the replication of HEV; however, the location of GBF1 is not changed upon HEV infection. GBFI does not co-localize with the ORF1 proteins of the virus, suggesting that GBF1 may not be recruited to the replication sites [101].

The mass spectrometry analysis of proteins interacting with the HEV RNA at its putative promoter region and the RdRp reveals a number of protein candidates, most of them localized to ribonucleoprotein granules, secretory granule lumen, and endocytic vesicle lumen [149]. The functional category of the proteins includes translation elongation, RNA binding, and RNA stem-loop binding. The highest number of proteins are involved in nucleic acid-binding activity, which further confirms the dependence of HEV on the host for viral RNA metabolism [149].

A yeast hybrid assay against partial HEV capsid protein (aa 112–660) reveals 59 proteins involved in various biological processes, such as homeostatic process and oxidation. The major portion of these are membrane proteins associated with ER, Golgi, mitochondrion, and transportation vesicles [150].

BioID, a method to screen for physiologically relevant protein interactions in living cells, and mass spectrometry (BioID/MS) were conducted to determine the host proteins in close proximity to the capsid protein of a genotype 3 strain [133]. A total of 145 potential proximal interactors was identified. Gene ontology analysis shows that the capsid protein is associated with the mitochondrion proteins and ribonucleoprotein granules, suggesting that the capsid protein may interfere with some mitochondrial processes and has a role in the post-transcriptional regulation of cellular RNA. The ORF2 proximal interactors are highly enriched in proteins of RNA metabolism, regulation of translation, and proteolysis. The BioID/MS data analysis shows the top candidate is DDX3, suggesting a strong interaction with the capsid protein. Notably, DDX3 silencing led to a significant reduction in HEV replication [133]. The ATPase activity of DDX3 is also required for HEV replication. These results demonstrate a pro-viral role of DDX3 in HEV replication, providing further insights into the virus-cell interactions.

Recent protein-protein interaction analysis revealed that a series of translation initiation factors, such as eIF4A2, eIF3A, eEF1A1, and RACK1, are required for virus replication. Proteins assemble with viral nonstructural proteins like RdRp, X, and PCP, suggesting that the virus utilizes the cellular translation complex for viral protein translation [151].

The STAT3 transcription factor is a major regulator of the acute-phase response (APR) in the liver, which contributes to host defense [152]. The ORF3 product vp13 decreases the nuclear translocation of p-STAT3, possibly through the delayed post internalization trafficking of the epidermal growth factor receptor (EGFR) [153]. The delayed post internalization trafficking was later found to be mediated by the interaction between vp13 and CIN85, a multidomain adaptor protein implicated in the downregulation of receptor tyrosine kinases [154]. vp13 also interacts with α1-microglobulin/bikunin precursor (AMBP), an immune response suppressor, and promotes its cleavage and secretion out of hepatocytes [155]. The immune regulation may create an immune-tolerant environment for HEV replication.

Collectively, HEV can modulate host factors, such as lipid-associated molecules, translational factors, and immune components, to generate a conducive environment for replication. However, it is unclear whether different HEV genotypes modulate the host factors differently, leading to distinct disease manifestations. This variation may also be true for different strains with variable virulence in the same genotype. Further research is needed to compare the variable effects of the different genotypes, especially between genotype 1 causing acute infection with potential adverse outcome in pregnant women and genotype 3 causing chronic infection.

## 6. Clinical Manifestations of Hepatitis E

Common clinical signs of HEV infection include vomiting, nausea, fever, jaundice, and elevated liver enzyme [156]. Genotype 1 and 2 HEV mainly cause acute hepatitis after infection and are generally more virulent than genotype 3 and 4 in terms of causing diseases in the immunocompetent population [157]. Data from patients with confirmed acute HEV genotype 1 infection in England and Wales suggest that the incubation time ranges from 10–71 days, with a median of 29.8 days [158]. Viremia peaks within the incubation period or concurrent with the symptom onset [159,160] but can be prolonged in certain patients [161,162]. Viral RNA in the blood can become undetectable after three weeks from the disease onset, while the RNA in feces can be detected for a longer period [160].

HEV genotype 3 and 4 can infect both immune vulnerable and competent populations. Strains of these two genotypes can cause foodborne zoonotic infection and can be transmitted via uncooked or undercooked meat and products from swine, wild boars or deer [163]. The majority of cases caused by genotype 3 HEV are asymptomatic, while some cases in older adults and immunocompromised patients can result in chronic infection. The incubation period varies from 5–7 weeks after infection with a median period of 5.4 weeks, which is similar to genotype 1 [41]. In developed countries, HEV genotype 3 infection occurs mostly in the population around the age of 60 and older [157]. Infection of genotype 3 HEV in immunocompromised individuals bears a high risk of developing into chronic hepatitis, and in some cases the outcome can be accelerated cirrhosis, especially in organ transplant recipients [164,165,166]. Chronic infection with genotype 3 strains may last for 3–6 months after the onset of illness. Genetic heterogeneity analysis in 16 solid-organ-transplant patients showed that, after HEV infection, eight patients progressed to chronic hepatitis following the acute phase, and the ORF2 sequences from these eight patients possess greater diversity than others who cleared the virus during the acute phase. This higher genetic heterogeneity may help to evade immune surveillance and account for the disease chronicity [167]. Infection with genotype 4 is mostly autochthonous [168,169], which may be asymptomatic or present typical liver dysfunctional signs, such as vomiting, nausea, jaundice, elevated alanine aminotransferase (ALT), and even ascites. The clinical manifestations and chronicity determinants of chronic HEV infection have been reviewed elsewhere [170]. Genotype 3 and 4 infection in pregnant women does not cause fatal consequences, based on current reports [171,172].

Aside from the typical liver symptoms, HEV infection is also implicated in neurological disorders, which distinguish HEV infection from other forms of hepatitis [173]. Neurological manifestations, such as Guillain-Barré syndrome [174], Bell’s palsy [175], and neuralgic amyotrophy [176], have been observed in both acute and chronic HEV-infected patients. HEV has been detected in cerebrospinal fluid [177]. In an HEV genotype 4-infected Mongolian gerbils, the virus is detected in the neuron, ependymal epithelium, and choroid plexus area [178]. The data suggest that HEV is able to pass the blood-brain barrier to invade the brain and spinal cord. In genotype 3 HEV infected patients with neurological disorders, most of them appear to suffer no severe hepatic failure. Neurological pain is more frequently observed in immunocompetent patients over 50 years old [179]. In a recent clinical study, among 141 cases of acute HEV infection in Southern Switzerland, 43 (30.4%) had neurological symptoms within six months [180]. In the 141 cases, 15 (10.6%) showed neuralgic amyotrophy, and 28 (19.8%) presented myalgia. All 15 patients with neuralgic amyotrophy were immunocompetent, and men have higher odds of developing it. It is recommended that patients with acute neurologic manifestations and aminotransferase abnormalities should be screened for HEV infection. The reason for HEV-associated neurological manifestations remains inconclusive. One possible reason could be the neurotropism of the circulating HEV. Other extra-hepatic manifestations like acute pancreatitis can be seen in HEV infection [181]. A plausible reason for virus-associated acute pancreatitis is the direct inflammation and destruction of pancreatic acinar cells by the virus. The extra-hepatic manifestations associated with HEV infection are reviewed elsewhere [182].

HEV infection of patients with ongoing hepatitis can potentially accelerate disease progression. Co-infection with HEV exacerbates the disease progression of the other viral forms of hepatitis [183]. Co-infection of HAV and HEV has been found in Poland, and patients with HAV are more prone to be exposed to HEV infection [181]. Distribution of the dual infection is widespread, such as in Egypt [184], Iran [185], South Korea [186], and Italy [187]. The prevalence of co-infection is predominant towards the end of monsoons and the beginning of the winter in India [188]. HEV co-infections in chronic hepatitis B patients are also reported [189,190]. A clinical study shows that the co-infection of HBV and HEV has exacerbated complications and liver failure [183]. HEV infection causes severe adverse outcomes in both cirrhotic and non-cirrhotic chronic hepatitis B patients [191,192]. For patients with HBV and HEV co-infections, anti-HBV treatment does not decrease the mortality rate or improve the prognosis of liver failure [183]. In patients with chronic HCV infection who receive liver transplantation in the US, a high prevalence of HEV is detected [193]. The co-infection of HCV and HEV is also reported in China [194] and may exacerbate the disease progression.

HEV infection in animals is generally subclinical except in chickens. Avian HEV is associated with the hepatitis-splenomegaly syndrome, a disease with big liver and spleen in chickens [195], yet the majority of the infected chickens are subclinical.

## 7. HEV Infection of Pregnant Women

HEV infection during pregnancy can result in poor prognosis [196] and vertical transmission. Unlike HBV and HCV, which normally do not cause death cases in acute infection of pregnant women [197,198], HEV infection by genotype 1 strains in pregnant women results in up to 30% case fatality, while infection by genotype 3 strains is usually subclinical [3].

A fine balance is maintained during pregnancy between maternal-fetal tolerance and mounting an effective innate and adaptive immunity against invading pathogens. Dysregulation of the mechanisms maintaining the balance can lead to disorders. Early studies show that the number of T cells decreases during pregnancy [199,200,201]. There is a clear shift of the Th1:Th2 paradigm with an apparent Th2 bias, which could create maternal-fetal tolerance for fetus development [202]. Regulatory T cells are also considered to play a role in maternal-fetal tolerance during pregnancy [203]. Th2 bias in pregnant women with acute hepatitis E due to genotype 1 HEV is more prominent than in non-pregnant patients with acute hepatitis E and healthy pregnant women [204]. The obvious Th2 alteration might attribute to the physiological changes of pregnancy or the consequence of virus infection. These data suggest that genotype 1 HEV might be able to dysregulate the balance between tolerance and immunity.

Early antiviral response is critical for the host to control virus infection. In pregnant women with acute infection of genotype 1 HEV, the expression of TLR3, 7, and 9 in monocytes and macrophages is upregulated. But when acute liver failure (ALF) develops, their expression is reduced, which may account for the defective immune response in ALF patients [205]. Also, the IFN response in placental cells is reported to be weaker than hepatocytes for genotype 1 but not genotype 3 HEV [206]. These data suggest that genotype 1 HEV might be able to evade the early antiviral response in a certain population of pregnant women and cause an adverse consequence.

Genotype 1 HEV infection might interfere with adaptive immunity in some subjects. One study shows that in the second and third trimester of pregnancy, HEV-infected pregnant women with fulminant hepatic failure (FHF) have lower CD4^+^ T cell counts and higher CD8^+^ T cell counts than HEV-negative women with FHF [207]. Other studies show a significant infiltration of activated CD8^+^ T cells containing granzymes in liver biopsies from HEV-infected patients with FHF, which suggests the role of CD8^+^ T cells in the liver injury [113,114]. Clinical investigation in HEV-infected women with acute liver failure revealed that the cytokine level, such as TNF-α, IL-6, and IFN-γ, is higher than in non-infected pregnant women, and the level of these cytokines are positively correlated to adverse pregnancy outcome [136]. These data suggest that genotype 1 HEV induces an overactive inflammatory response leading to a poor prognosis in a certain population of pregnant women.

The genotype 1 HEV is also implicated in invading the placenta more effectively than genotype 3. A recent study demonstrates that the genotype 1 HEV grows much more dynamically than genotype 3 HEV in the ex vivo maternal-fetal interface model using the decidua basalis and fetal placenta [208]. Genotype 1 HEV induces a higher level of apoptosis of decidua and placenta cells than genotype 3 HEV and provokes a higher level of IL-6, CCL-3, and CCL-4, which positively correlates with the viral load. Meanwhile, UV-treated culture supernatant harvested from genotype 1 HEV-infected explants causes more tissue injury in fresh decidua and placenta organ culture than the genotype 3 HEV-infected cultures [208]. Further studies are needed to compare the genetic characteristics of genotype 1 and 3, and identify the key factors in genotype 1 infection that determine severe disease outcome.

In addition to immunological factors, hormone variation during pregnancy may contribute to viral replication. Pregnant women with acute HEV hepatitis have increased estrogen and progesterone level [207]. These hormones are known to dampen the cell-mediated immune response [209,210]

Similarly, the estradiol level in HEV-infected pregnant women during the third trimester is significantly higher than HEV-negative pregnant women. An in vitro experiment shows that the estradiol treatment facilitates HEV replication in A549 cells [211]. Thus, the variation in hormone level is also speculated to be one of the factors that contribute to the severe disease progress of HEV-infected pregnant women.

Collectively, the immune-tolerant environment may subject pregnant women to become more vulnerable to HEV infection. HEV infection with genotype 1 might dysregulate the balance maintaining the maternal-fetal tolerance and effective immunity, or alter the immunological mechanisms that maintain the balance, which leads to the dire consequence of FHF in pregnant women. The potential mechanisms that genotype 1 HEV employs to produce the mortality of pregnant women need to be investigated. Why infection of different genotypes results in different disease outcomes and what determines the chronicity of HEV infection remain unclear. Both virus and host factors possibly contribute to the disease’s progress. In terms of the viral factors, genetic variations of the different genotypes and host adaptability may account for the different outcomes. Conversely, the immune-tolerant environment and associated factors that may be better exploited by genotype 1 HEV in a certain vulnerable population might contribute to the development of ALF in infected pregnant women. So far, most fatality cases of HEV infection during pregnancy are found in South Asia. Due to the different geographical environments and food habits, the gut microbiota in South Asia might vary from other areas and might be a risk factor for ALF development in HEV-infected pregnant women. Further research on this front is needed to address this speculation.

## 8. Concluding Remarks

HEV infection causes large outbreaks of hepatitis across the world. In pregnant women or patients with immunosuppression, the prognosis of HEV infection can be poor. The reason that genotype 1 HEV causes mortality in pregnant women needs to be further investigated. Infection of different genotypes results in different disease outcomes, and the determinants of the chronicity of the HEV infection remain unclear. Both the virus and host factors can contribute to the process. In terms of the virus factor, the genetic variations and host adaptability may account for the different symptoms. In terms of host factors, the compromised immune system may lead to FHF development in HEV-infected pregnant women or provide a tolerant environment for persistent infection.

In the virus life cycle, for the formation of eHEV in the cells the virus acquires the envelope mainly from the intracellular membrane. It is not known whether HEV alters the process of the host fatty acid metabolism for its replication and whether the lipoproteins are employed in the viral life cycle. The presence of a lipid envelope lowers the entry efficiency of the virus. Although HSPG is demonstrably required for HEV entry, the specific receptors for non-enveloped or enveloped HEV remain to be elucidated. Host factors are essential for HEV replication, and the complex virus-cell interactions lead to a conducive environment for the viral proliferation. There remain many unknowns in this process.

Recent studies assist the understanding of HEV biology and pathogenesis. However, there are still many open questions. For instance, what is the receptor for the viral entry? What is the location of the viral replication complex? What are the cellular factors that HEV manipulates to generate a conducive environment for replication? How does the virus recruit the cellular factors for its replication? Does HEV influence liver lipid metabolism? What are the roles of different forms of the capsid protein? What is the role of the quasi-enveloped virions in HEV transmission? What are the differences between genotype 1 and 3 that account for the difference in host range and disease outcomes? Are there any host factors or other conditions in a certain population of pregnant women in South Asia that are prone to the genotype 1 HEV-induced FHF? In order to answer these questions, an efficient cell culture system and a competent animal model, as well as specific antibodies against individual viral proteins, are needed. A recent report in cell culture for high yield of HEV [212] and the rabbit model for HEV-induced adverse pregnancy outcome [213] will be useful to address some of these questions. Further study on HEV biology and pathogenesis is warranted.

## Figures and Tables

**Table 1:** Summary of the forms of ORF2 product [6].

Form ^a^	Start Codon	Genome Association	Glycosylation	Secretion
ORF2c	Met15	Yes	No	No
ORF2s	Met1	No	Yes	Yes

^a^ ORF2c: the actual capsid protein, ORF2s: the secreted ORF2 product.

## Data Availability

Not applicable.
